# Refined Automatic Brain Tumor Classification Using Hybrid Convolutional Neural Networks for MRI Scans

**DOI:** 10.3390/diagnostics13050864

**Published:** 2023-02-23

**Authors:** Fatma E. AlTahhan, Ghada A. Khouqeer, Sarmad Saadi, Ahmed Elgarayhi, Mohammed Sallah

**Affiliations:** 1 Mathematics Department, Faculty of Science, Mansoura University, Mansoura 35516, Egypt; 2 Physics Department, Faculty of science, Imam Mohammad Ibn Saud Islamic University, Riyadh 11564, Saudi Arabia; 3 Applied Mathematical Physics Research Group, Physics Department, Faculty of Science, Mansoura University, Mansoura 35516, Egypt; 4 Higher Institute of Engineering and Technology, New Damietta 34517, Egypt

**Keywords:** brain tumors classification, T1 MRI scans, GoogleNet CNN, AlexNet CNN, support vector machine (SVM), K-nearest neighbor (KNN)

## Abstract

Refined hybrid convolutional neural networks are proposed in this work for classifying brain tumor classes based on MRI scans. A dataset of 2880 T1-weighted contrast-enhanced MRI brain scans are used. The dataset contains three main classes of brain tumors: gliomas, meningiomas, and pituitary tumors, as well as a class of no tumors. Firstly, two pre-trained, fine-tuned convolutional neural networks, GoogleNet and AlexNet, were used for classification process, with validation and classification accuracy being 91.5% and 90.21%, respectively. Then, to improving the performance of the fine-tuning AlexNet, two hybrid networks (AlexNet-SVM and AlexNet-KNN) were applied. These hybrid networks achieved 96.9% and 98.6% validation and accuracy, respectively. Thus, the hybrid network AlexNet-KNN was shown to be able to apply the classification process of the present data with high accuracy. After exporting these networks, a selected dataset was employed for testing process, yielding accuracies of 88%, 85%, 95%, and 97% for the fine-tuned GoogleNet, the fine-tuned AlexNet, AlexNet-SVM, and AlexNet-KNN, respectively. The proposed system would help for automatic detection and classification of the brain tumor from the MRI scans and safe the time for the clinical diagnosis.

## 1. Introduction

Among the organs in the human body, the human brain is considered one of the most important and complex organs [1]. Its neural cells along with the supporting ones help the carry out the functions and activities normally [1,2]. The cells of human brain are considered as the main controller for many activities such as emotion, muscles movement, memory, vision, temperature, breathing, and all processes that regulate our body [2]. Each cell in the human brain specializes in specific tasks, and any defect in its functions may lead to serious diseases [3]. The brain tumor is considered one of the most life-threatening and aggressive diseases, whether it be in adults or children [3,4].

Brain tumors are masses or collections of abnormal cells proliferating uncontrollably [1,4]. They can lead to death if type-specific appropriate treatment is not initiated promptly [5]. In 2021, approximately 83,570 patients were diagnosed with brain tumors in the USA (59,040 nonmalignant tumors and 24,530 malignant tumors), with approximately 18,600 dying from brain cancers [6].

Brain tumors can be divided into two main classes: benign (non-cancerous) and malignant (cancerous) tumors [1,4,5]. Benign tumors are usually characterized by a slow, controlled rate of growth with possible encapsulation. Therefore, this type of tumor can compress different regions of the brain, depending on its location. Nearly all benign tumors do not spread or metastasize to other parts of the body, and are considered with less medical risk than the malignant [2,3]. Meningiomas and pituitary tumors are considered the most common types of benign tumors [6]. Meningiomas originate from the thin membranes that cover the spinal cord and brain [1,5]. On the other hand, pituitary tumors originate from the pituitary gland at the base of the brain [3,6].

On the other hand, the spread of malignant tumor is quick, and attacks other normal (healthy) cells leading to its growth in other parts of the human body [2]. Gliomas are considered the most common type of the malignant tumor, especially grades 3 and 4 [1,2,3,4]. Gliomas start in the spinal cord or brain; the group includes tumors such as oligoastrocytomas, astrocytomas, oligodendrogliomas, and glioblastomas [2]. Due to their highly sensitive locations and associated morbidity and mortality, malignant tumors also need targeted appropriate treatment. This is highly dependent on the type of tumor since each type has its own established protocols [2,5]. Recently, interest has grown in using artificial intelligence (AI), specifically Machine Learning (ML), and Deep Learning (DL), to detect the tumor type and reduce misdiagnosis risk.

DL, a subfield of ML, is considered one of the remarkable computational intelligence techniques [7,8]. It can enable computers to make forecasts and create conclusions based on historical data [8,9]. As such, DL algorithms have found many potential uses in various fields and significant success in medical imaging classification and automatic diagnosis [5]. Training a network on a big dataset and then transferring the learned network to a small dataset is known as transfer learning of DL categories [7,8,9,10,11]. There are two methods for transfer learning; either fine-tuning the ConvNet, or freezing the layers of ConvNet [12]. Fine-tuning takes place through replacing and retaining the pre-trained ConvNet on the target dataset to apply back-propagation [10,11]. Then the target dataset may be classified through the last fully connected layer [9]. Nevertheless, the last fully connected layers are removed since they act as features passed to a classifier such as SVM, KNN, or Naïve Bayes to complete classification process [13].

## 2. Related Work

Various AI strategies are proposed for detecting and classifying brain tumor types via magnetic resonance imaging (MRI) scans [14,15,16,17,18,19]. In 2015, Cheng et al. [14] presented a method to enhance brain tumor classification by using the principles of partition and augmentation. They utilized Gray-Level Co-occurrence Matrix (GLCM) for extracting the features of brain tumor scans. The highest accuracy reported by this method was 91.28%.

Ismael et al. [15] used the Discrete Wavelet Transform (DWT) method for extracting brain tumor features from MRI slices. Then, they employed a back-propagation multilayer perceptron neural network for training and classifying the features. They achieved an accuracy reaching 91.9%. Capsule Networks (CapsNets) were suggested by Afshar et al. [16] for detecting the types of brain tumors. They extracted 64 feature maps from only one conventional layer with accuracy 86.56%.

Abir et al. [17] conducted a classification experiment for classifying brain tumor classes; they used the GLCM to extract the features and a Probabilistic Neural Network (PNN) for training and classifying these features. The highest accuracy obtained by this method was 83.33%. Abiwinanda et al. [18] deployed a CNN including two convolutional layers, max-pool, and ReLU layer followed by 64 hidden neurons. The obtained training and validation accuracies achieved by this network were 98.51% and 84.19%, respectively. Chattopadhyay and Maitra [19] introduced an MRI-based brain tumor image-detection technique using a DL method. While achieving an incredible accuracy of 99.74%, their database consisted of only two labels, tumor and non-tumor, neglecting all the types discussed previously [19].

However, brain tumors are one of the most often diagnosed malignant tumors in the head and neck and recognizing their grade is challenging for radiologists; it is critical to detect and classify contaminated tumor locations using MRI scans. Therefore, Vankdothu et al. [20] introduced a CNN for detecting brain tumors using MRI images. However, there were still issues with the training length, leading them to develop an IoT computational system based on DL for detecting brain tumors in MRI images. They suggested combining CNNs with the Long Short Term Memory and performed experiments on a Kaggle dataset with 3264 MRI scans to forecast the proposed model’s performance. The dataset was separated into 2870 photos for training and 394 images for testing. The experiment demonstrated that the proposed model outperforming the earlier CNN and RNN models in terms of accuracy.

Deepak and Ameer [21] used a CNN to classify brain tumor MRI scans using multiclass brain tumor datasets. They compared results of classifiers such as Decision Tree (DT), Naive Bayes (NB), Linear Discrimination (LD), K-nearest Neighbor, and Support Vector Machine (SVM). They presented an automatic classification system designed for three brain tumor types: glioma, meningioma, and pituitary brain tumors. A comprehensive evaluation of the proposed system was conducted to record the best classification performance. They could produce acceptable results with a smaller number of training samples.

Konwar et al. [22] proposed a method for automated brain tumor segmentation using U-Net deep convolutional networks. This method may help detect brain tumors in the early stages. For correct tumors’ extent evaluation, they developed a reliable fully automated brain tumors segmentation approach, presenting a completely automated brain tumors segmentation system using the Multimodal Brain Tumors Image Segmentation (BRATS 2015) datasets to test their approach.

Haq et al. [23] proposed a robust brain tumor classification DL method to improve the accuracy of existing artificial diagnosis systems. They used an improved convolution neural network to classify brain tumors based on cranial MRI scans. Their model’s classification performance improved by incorporating data augmentation and transfer learning methods, having obtained high accuracy compared to the baseline models; henceforth, they suggested incorporating the proposed model for brain tumor diagnoses in IoT-healthcare systems.

Rao et al. [24] proposed a brain tumor detection approach using a deep learning-based technique. They presented a modified U-Net structure based on residual networks that uses sub-pixel convolution at the decoder part and periodic shuffling at the encoder section of the original U-Net. The advantage of sub-pixel convolution over traditional resizing convolution is that it contains additional parameters, which results in higher modeling capabilities at the same processing complexity and prevents de-convolution overlapping. On two benchmark datasets, the proposed U-Net model was characterized by segmentation accuracies of 93.40% and 92.20%, respectively. Additionally, three categories—tumor core, total tumor, and augmenting core—were created from the classification of the tumor sub-regions. The test results showed that the recommended U-Net performed better than current methods.

A categorization strategy for brain tumors based on a CNN was suggested by ZainEldin et al. [25]. It was proposed as an adaptive dynamic sine–cosine fitness grey wolf optimizer technique for CNN hyperparameter optimization. Following the hyperparameter tuning, an Inception-ResnetV2 training model was created. The model used frequently employed pre-trained models to enhance the diagnosis of brain tumors, and its output was binary, with 0 denoting normal and 1 denoting malignancy. Hyperparameters may be broadly divided into two categories: (i) those that specify the underlying network topology, and (ii) those that train the network. According to the experimental findings, the recommended CNN classifier produced the best results, since the hyperparameters optimization improved its performance, reaching an accuracy rate of 99.98%.

Therefore, the main goal of the current work is to enhance the performance of CNNs for detecting and classifying the brain tumors categories based on MRI scans. A composite database was generated from three other databases (Figshare, SARTAJ, and Br35h), yielding 2880 scans in total; these scans included the three main types of brain tumor (gliomas, meningiomas, and pituitary tumor) as well as normal ones with no tumor. Four refined CNNs are proposed for classifying brain tumor categories and tested using the compiled dataset. Testing and validation accuracy is presented using a confusion matrix.

## 3. Materials and Methods

### 3.1. Dataset of the Brain MRI Scans

**Figure 1:**
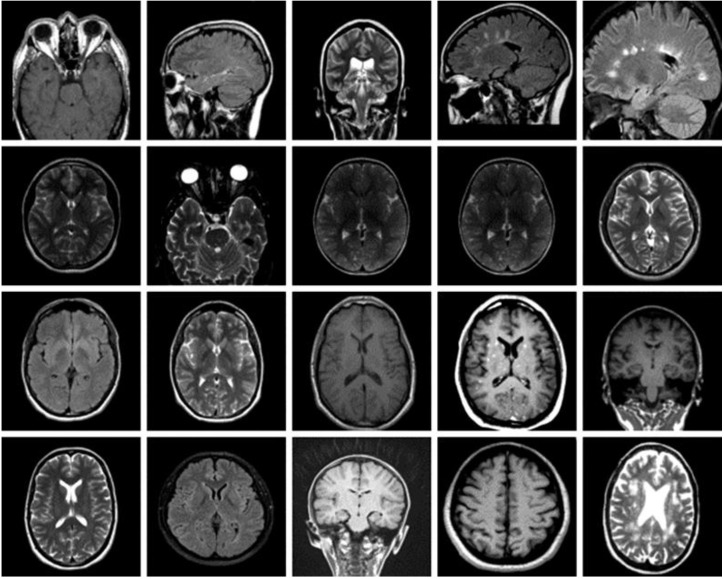
Examples of MRI scans with no tumor.

**Figure 2:**
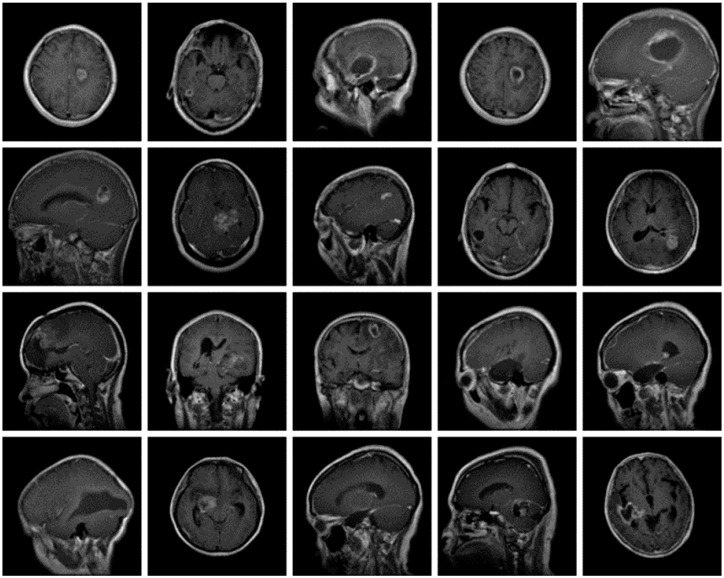
Examples of MRI scans of Glioma tumors.

**Figure 3:**
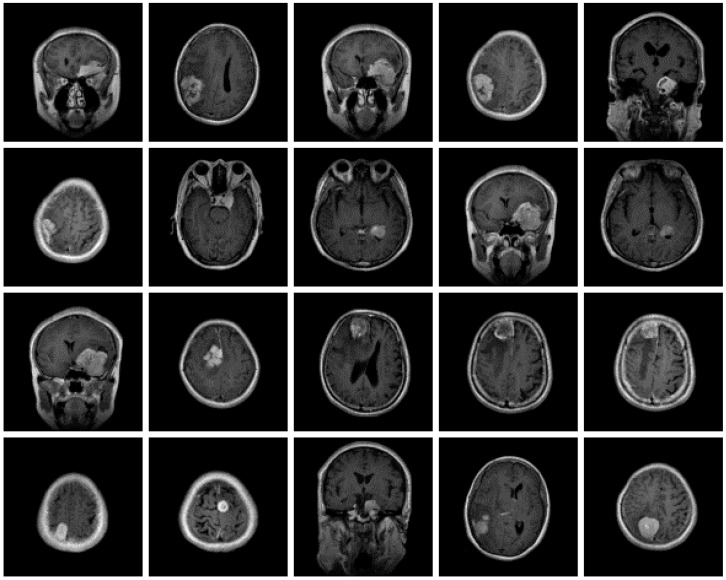
Examples of MRI scans of meningioma tumors.

**Figure 4:**
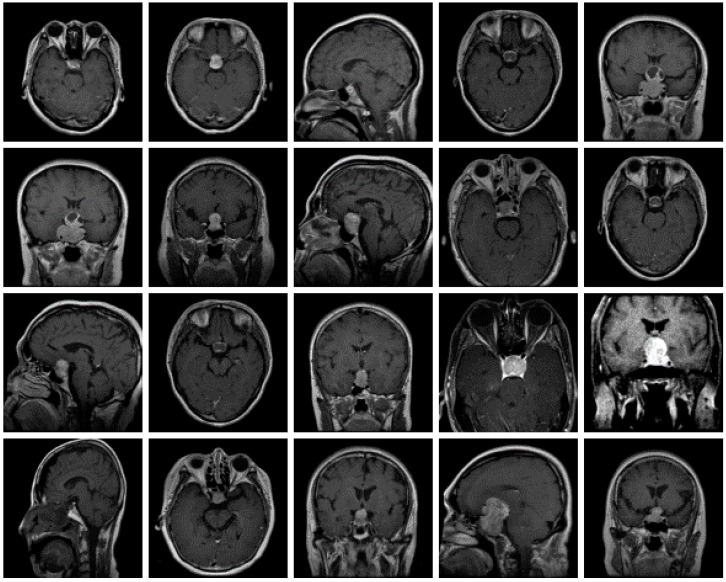
Examples of MRI scans of pituitary tumors.

The dataset used for this current work is compiled from three other datasets: Figshare, SARTAJ, and Br35h. This composite dataset contains 2880 T1- weighted contrast-enhanced MRI brain images [26]. Gadolinium was used as contrast agent in these images. The target dataset has 829 images for glioma tumor, 825 images for meningioma tumor, 830 images for pituitary tumor and 396 images for no tumor cases. The images of the target dataset have a resolution of 512 × 512 × 3 with 24 bits color depth. Figure 1, Figure 2, Figure 3 and Figure 4 show some examples for each possible class: no tumor, glioma, meningioma, and pituitary tumor, respectively. The target dataset is split with 70% used for training and the remaining 30% kept aside for estimating the validation accuracy.

This spliting was performed using Matlab’s function “splitEachLabel”, which allows specifying both the training and validation proportions. Moreover, the KFold partition data technique and Random sampling method were also employed.

### 3.2. The Architectures of the Proposed CNNs

**Figure 5:**
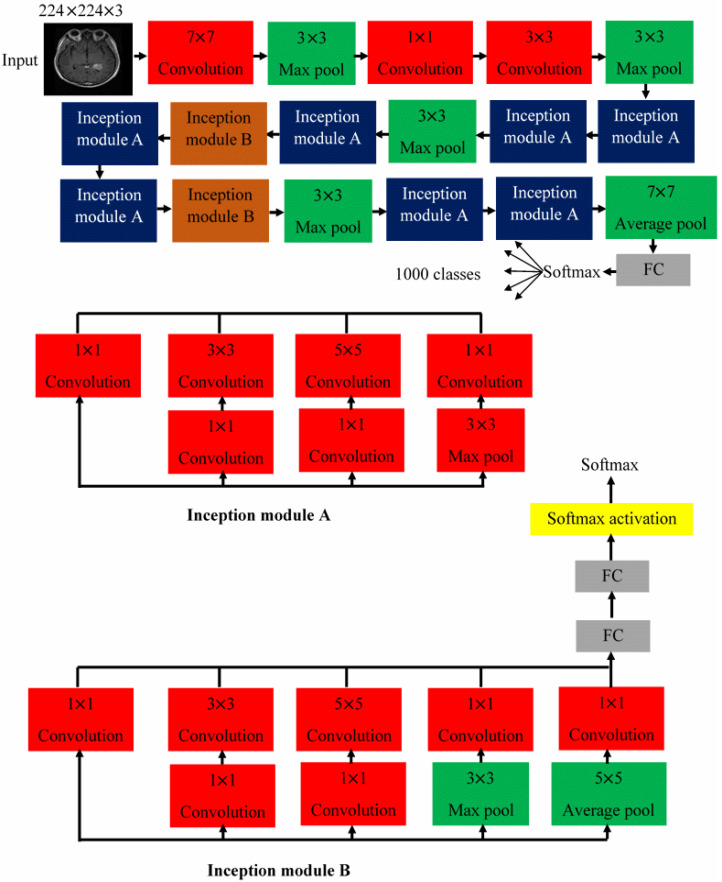
The architecture of the pre-trained GoogleNet.

In this work, the GoogleNet and AlexNet networks were employed for training and classifying the MRI images. Google introduced the inception structure GoogleNet at the 2014 ImageNet Large-Scale Visual Recognition Challenge (ILSVRC14), being the best-performing model. GoogleNet is a convolutional neural network architecture, displayed in Figure 5 [27]. It uses Inception modules, permitting the network to choose between multiple convolutional filter sizes in each block [9]. Its construction uses methods such as global average pooling and a 1 × 1 convolution and that allows it to create deeper architecture to raise the performance of the neural network [5]. As such, when the layer becomes deeper, computational complexity grows exponentially [5,27]. The input data are fed into four independent layers (1 × 1, 3 × 3, 5 × 5 convolution layers and 3 × 3 max pooling layer), and the outputs are combined into a single dataset [5,8].

Inside, the convolutional layers extract various spatial features. The process of extracting distinct features is the role of information of the input data and the maximum pooling layer. This reduces the channel and size of the input data [27]. Thus, the inception module extracts more information into a smaller layer by growth the layer of the neuron network, which is only composed of the existing depth [9,27]. GoogleNet accepts input images of size 224 × 224 × 3 [9].

**Figure 6:**
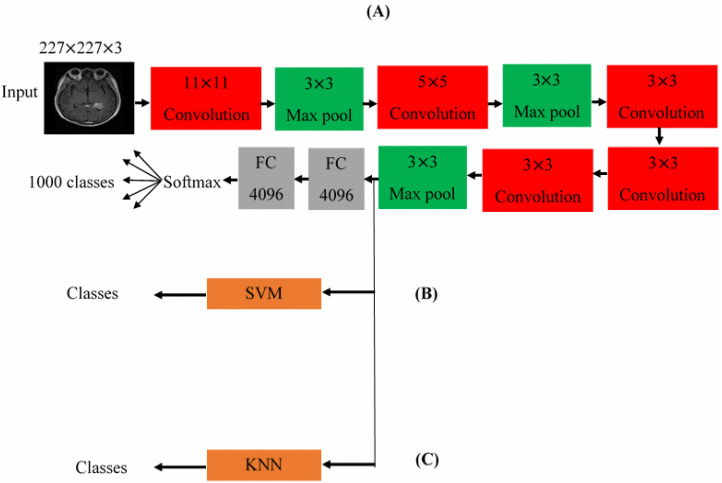
The architectures for the pre-trained (**A**) AlexNet, (**B**) hybrid AlexNet SVM, and (**C**) the hybrid AlexNet KNN.

Krizhevsky et al. [28] proposed the AlexNet model in 2012. It has eight layers with learnable parameters [28,29]. It is composed of five layers with a combination of max pooling as shown in Figure 6A [28]. Three fully connected layers come after, each apart from the output layer, use ReLu activation. ReLu works as an activation function, which can hurry the training process by a factor of six. The AlexNet is trained on the ImageNet dataset, which has almost 14 million images and 1000 classes [28,29]. AlexNet accepts input images of size 227 × 227 × 3 [29]. In this work, we have applied the convolutional neural network as an automatic feature extractor through its layers; hence, no image segmentation was needed.

## 4. Experimental Results and Analysis

### 4.1. Classification the Brain Tumor Using CNNs

Throughout this work, the pre-trained GoogleNet and AlexNet were used as transfer learning networks for extracting MRI scan features for the different brain tumors; these features were then used for classification training. The GoogleNet and AlexNet cannot be used directly for classifying the images as the original settings and parameters for both are optimized for classifying one thousand categories, compared to the four categories in the current dataset. Moreover, the receptive field size of the target dataset (512 × 512 × 3) is quite different from the receptive field sizes of Alexnet (227 × 227 × 3) and GoogleNet (224 × 224 × 3). Therefore, the original settings and parameters of the GoogleNet and the AlexNet had to be fine-tuned before the classification task. Here, the input data represents the features extracted from the images fed to the Net; the classification accuracy depends on what characteristics are used, with some being more useful for the classification process, and others reducing the accuracy. Due to the network layers’ ability to extract the most useful distinguishing characteristics, this helps improve performance and obtain better results.

In GoogleNet, the fine-tuning process can be implemented by replacing its last two learnable layers, utilized for retrieving the brain tumor features, and its final softmax layer with new layers that include a number of neurons adjusted to the number of classes in the target dataset (four classes). Additionally, resizing the dimensions of the brain tumor images in the target dataset from 512 × 512 × 3 to 224 × 224 × 3. This concluded the GoogleNet parameters and conditions adjustment, making it now reading for tumor classification.

**Figure 7:**
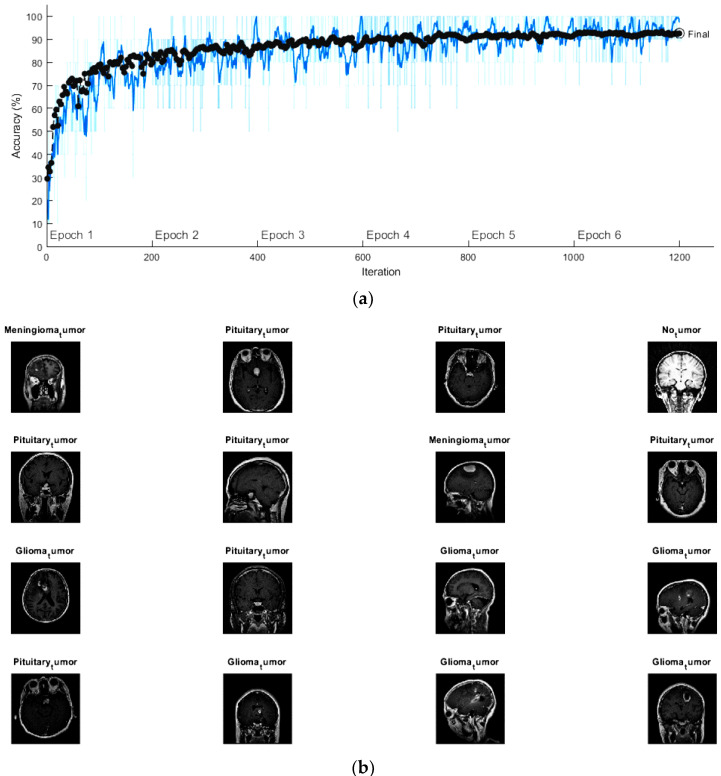
(**a**) The progress graph of the pre-trained GoogleNet for the brain tumors and (**b**) some validated brain tumors with their predicted labels using fine-tuning GoogleNet.

The initial learning rate, the maximum epochs and the minimum batch size of the fine-tuned GoogleNet were set to be 10-4, 6, and 10, respectively. In addition, the activation function used for this network was the stochastic gradient descent momentum (SGDM) function. Figure 7a shows the progress graph of this network using the training and validation sets. It became clear that the accuracy of the validation was reaching 91.5% after 1200 iterations with execution time totaling 4 h, 45 min, and 2 s. Several MRI scans from the validation set with the predicted labels are displayed in Figure 7b.

For fine-tuning AlexNet in order to be compatible with the number of classes in the target dataset, the last three layers (two FC layers the softmax layer) were exchanged with three new layers containing number of neurons, which is suitable for the current four classes. For matching the receptive field size of the brain tumor images with the receptive field size of AlexNet, all images in the target dataset were resized to 227 × 227 × 3.

**Figure 8:**
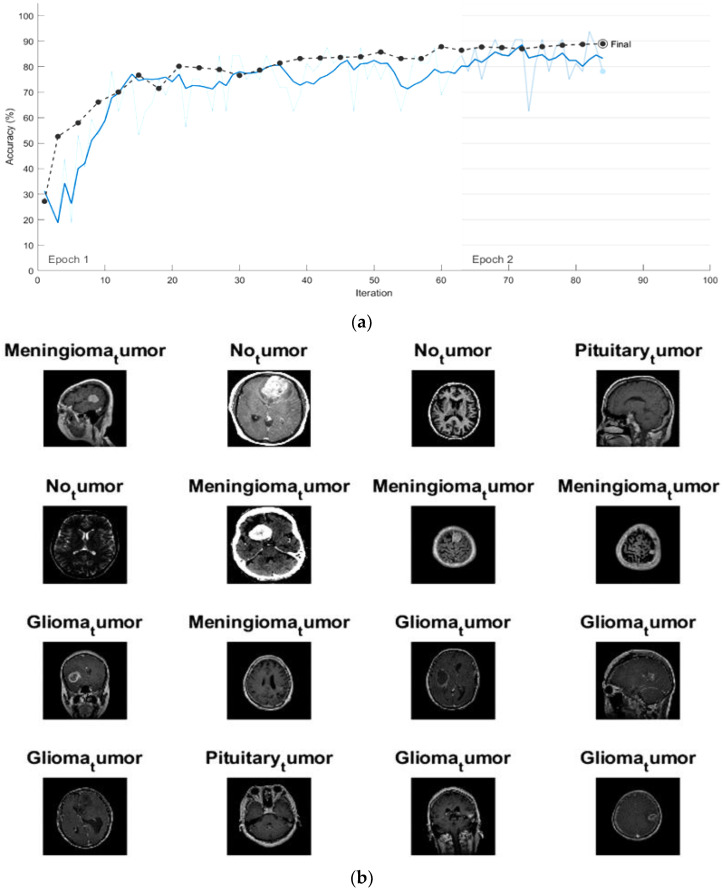
(**a**) The progress graph of the pre-trained AlexNet for the brain tumors and (**b**) some validated brain tumors with their predicted labels using fine-tuning AlexNet.

The same conditions and activation function that were used for GoogleNet training were utilized for AlexNet. The progress graph for accuracy and loss rates for the training and validation sets of the brain tumor images using the fine-tuning AlexNet is shown in Figure 8a. From this graph, the training process stopped after 88 iterations with execution time of 24 min and 12 s. Here, the determined validation accuracy was found to be 90.21%. Figure 8b shows some scans from the validation set with AlexNet’s predicted tumor label.

**Figure 9:**
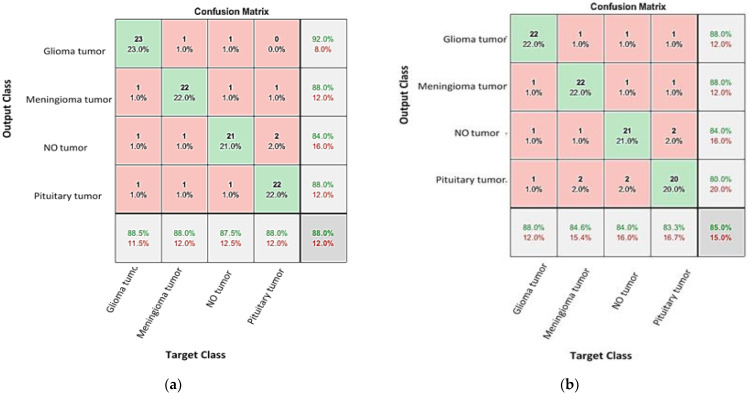
The confusion matrixes for testing brain tumors using the fine-tuning: (**a**) GoogleNet and (**b**) AlexNet networks.

For evaluating the testing accuracy of the fine-tuned GoogleNet and AlexNet, new smaller datasets, each including 100 brain tumor scans equally split among the four classes were used. After this, a confusion (error) matrix was generated to estimate the effectiveness of the suggested networks. Such a matrix computes and presents the testing accuracy, precession, recall, specificity, and F1-Score of a model as follows [29,30,31,32,33,34]

(1) $$\mathrm{Accuracy}\left(\%\right)=\frac{TP+TN}{TP+TN+FP+FN}\times 100,$$

(2) $$\mathrm{Precession}\left(\%\right)=\frac{TP}{TP+FP}\times 100,$$

(3) $$\mathrm{Recall}\left(\%\right)=\frac{TP}{TP+FN}\times 100,$$

(4) $$\mathrm{Specificity}\left(\%\right)=\frac{TN}{TN+FP}\times 100,$$

(5) $$F1-\mathrm{Score}\left(\%\right)=\frac{2\left(\mathrm{Precession}\times \mathrm{Recall}\right)}{\mathrm{Precession}+\mathrm{Recall}}\times 100$$

where *TP*, *TN*, *FP*, and *FN* represent the true positives, true negatives, false positives, and false negatives, respectively. Figure 9 displays the confusion matrices for the validation images when fed into the fine-tuned GoogleNet and AlexNet networks, respectively. From these matrices and using relation (1) above, the accuracy of the two networks were 88% and 85%, respectively. Hence, it is concluded that the accuracy of validation and testing for both networks are relatively low and cannot be reliably used to determine the brain tumor classes.

### 4.2. Enhancing the Classification Performance of the Fine-Tuning AlexNet for the Brain Tumor Classes Using Hybrid Networks

Two hybrid networks (AlexNet-SVM and AlexNet-KNN) are proposed for improving the accuracy of validation and testing for the fine-tuned AlexNet. The first proposed hybrid network is AlexNet-SVM, which consists of the first five convolutional layers of AlexNet and a support vector machine (SVM) as a classifier. The structure of this network is shown in Figure 6B. Here, the convolutional layers of the AlexNet are utilized for normalizing and extracting the features of the brain tumor images while an SVM classifier is trained on these features by building up an N-dimensional optimal hyperplane [31].

**Figure 10:**
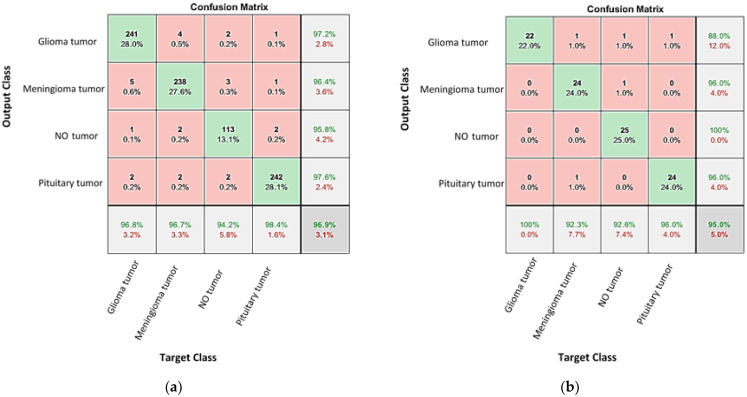
The confusion matrixes for the tested and validated brain tumors using the AlexNet-SVM networks for (**a**) validated and (**b**) tested brain tumor images.

This hyperplane works to maximize the edge between the data point classes [30,31]. For the current network, cross validation was run at five folds for training and classifying the extracted features. Figure 10a shows the confusion matrix for the hybrid AlexNet-SVM network on the training data. Using Equation (1), the accuracy of validation of this network is 96.9%. After exporting this network, the small dataset is used to explore its testing accuracy. Similarly, Figure 10b shows the confusion matrix for the network on the validation set, yielding a testing accuracy is estimated using relation (1) and found to be 95%.

The second proposed hybrid network is an AlexNet-KNN. Similarly, it employed the convolutional layers of AlexNet for normalizing and retrieving the features while a K-nearest neighbor (KNN) classifier was used for training and classification. The structure of this network is shown in Figure 6C. The KNN classifier is the simplest machine leaning classifier which utilizes distance metric functions (such as Euclidean distance, Minkowski distance, Manhattan distance, cosine Distance, and others), as well as a prespecified number of nearest neighbors (K = 1, 3, 5……) for determining the distance between any two points [35].

**Figure 11:**
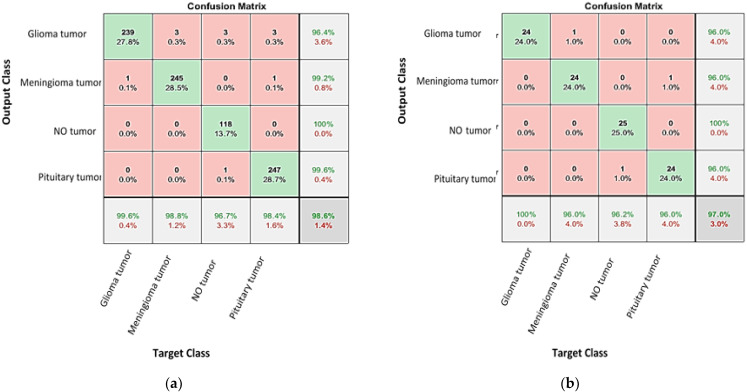
The confusion matrixes for the tested and validated brain tumors using the AlexNet-KNN networks for (**a**) validated and (**b**) tested brain tumor images.

When classifying a new data point, a KNN classifier functions by finding the closest instance that has approximate similar distance [30]. In the current work, the hybrid network AlexNet-KNN was established with cross validation folds set to 5, K = 5, and Minkowski distance as the distance function. Figure 11a shows the confusion matrix for the AlexNet-KKN network on the training set, yielding a validation accuracy of 98.6%. After exporting the network and testing it, Figure 11b shows the confusion matrix that. From it, a testing accuracy of 97% is calculated.

The above results show that the proposed AlexNet-KKN network has a higher validation and testing accuracy compared to the fine-tuned GoogleNet, fine-tuned AlexNet, and AlexNet-SVM. All the results from the confusion matrices are displayed in Table 1. In addition, the precession, recall, specificity, and F1-Score are calculated for each network and tabulated in Table 1, to verify the results.

The area under the curve (AUC) is calculated for the AlexNet-KNN network and found to be 0.99. To illustrate the advantages of the proposed method, the current work is compared to similar research in open literatures as shown in Table 2. It shows that the proposed method is unique in its high accuracy and ability to classify the largest number of brain tumor types compared to other published works. Moreover, the presented algorithm can extract features automatically, as opposed to other techniques in which characteristics are extracted using methods such as segmentation. This has the potential of aiding doctors with diagnosing brain tumor subtypes with high accuracy, and thus reducing the possibility of diagnostic errors. The obtained accuracy depends on multiple parameters such as the learning rate, activation function and k-fold, which need to optimized during the training process.

## 5. Conclusions

In this study, pioneering transfer learning and hybrid convolutional neural networks were suggested for classifying brain tumors scans with a high degree of accuracy. A dataset consisting of 2880 T1-weighted contrast-enhanced MRI brain images was used, each one containing four classes (no tumor, glioma, meningioma and pituitary tumor). The transfer learning techniques were employed for fine-tuning two CNNs (GoogleNet and AlexNet) and classifying the brain tumor classes. Initial results of validity and test accuracy were not encouraging; therefore, two other networks (AlexNet-SVM and AlexNet-KKN) were conceived, each incorporating CNNs with a machine learning classifier. The highest accuracy 98.6% was that of the hybrid AlexNet-KKN network. All in all, this showed the decisions of the AlexNet-KKN network to be reliable for determining and classifying brain tumor scans. 

## Figures and Tables

**Table 1:** Results of the performance for the suggested networks.

Network	Validation (%) ^1^	Testing Accuracy (%)	Precession (%)	Recall (%)	Specificity (%)	F1-Score (%)
GoogleNet	91.5	84–92	88.46	88	96.05	88.46
AlexNet	90.2	80–88	88	84.62	95.95	86.27
AlexNet-SVM	96.9	88–100	88	100	96.15	93.62
AlexNet-KNN	98.6	96–100	96	100	98.68	97.96

^1^ Highest validation accuracy.

**Table 2:** Comparison of the proposed method with published work.

Reference	Tumor Classes	The Used Classifier Model	Accuracy
B. Srinivas et al. [36]	Malign and Benign	CNN-KNN	96.25%
Nawab et al. [37]	Glioma, meningioma, and pituitary	Block-wise transfer learning	94.82%.
Mircea et al. [38]	Benign or low-grade (1, 2) and malignant or high-grade (3, 4)	Wavelet transforms and support vector machines	91%
F. Özyurt et al. [39]	Malign and Benign	NS-EMFSE–CNN–(KNN & SVM)	90.62% 95.62%
M. Sajjad et al. [40]	Benign or low-grade (1, 2) and malignant or high-grade (3, 4)	Fine-tune- VGG-19/Softmax classifer	90.67%.
K. Salçin [41]	Glioma, meningioma, and pituitary	Faster R-CNN	91.66%
J. Amine et al. [42]	Benign or low-grade (1, 2) and malignant or high-grade (3, 4)	Inception-V3 and DensNet201	89%
Our work	Glioma, meningioma, pituitary, and no tumor cases	AlexNet-KNN	98.6%

## Data Availability

The dataset is available in Ref. [26].
